# From magma ocean to core–mantle boundary: the key role of hydrous ferropericlase in shaping deep Earth’s low-velocity anomalies

**DOI:** 10.1093/nsr/nwag258

**Published:** 2026-05-01

**Authors:** Shi-Dong Guan, Yu Wang, Zhi-Xue Du, Ya-Nan Yang, Yi-Gang Xu

**Affiliations:** State Key Laboratory of Deep Earth Processes and Resources, Guangzhou Institute of Geochemistry, Chinese Academy of Sciences, China; College of Earth and Planetary Sciences, University of Chinese Academy of Sciences, China; State Key Laboratory of Deep Earth Processes and Resources, Guangzhou Institute of Geochemistry, Chinese Academy of Sciences, China; State Key Laboratory of Deep Earth Processes and Resources, Guangzhou Institute of Geochemistry, Chinese Academy of Sciences, China; Department of Chemistry and Molecular Biology, University of Gothenburg, Sweden; State Key Laboratory of Deep Earth Processes and Resources, Guangzhou Institute of Geochemistry, Chinese Academy of Sciences, China

## Abstract

HPHT experiments assisted with NanoSIMS demonstrate that hydrous ferropericlase, stable in the early basal magma ocean, is an efficient water reservoir and may provide a new explanation for the origin of Earth's deep low-velocity anomalies.

Water is essential for Earth’s habitability, with its influence extending far beyond the surface to the planet’s deep interior. The preservation mechanism of deep-Earth water and its geophysical effects are key to understanding the planet’s internal evolution. Diamond inclusions originating from deep mantle sources and multiple isotopic signatures indicate the presence of primordial water [1], suggesting that hydrous domains in the deep mantle are linked to early-Earth processes—particularly the crystallization of a primordial magma ocean. Notably, this deep water reservoir is intimately associated with Earth’s low-velocity seismic structures: the large low shear velocity provinces (LLSVPs) and the ultra-low velocity zones (ULVZs).

Ocean-island basalts (OIBs), which are thought to sample the LLSVPs, contain ∼0.2–1.6 times the water content of mid-ocean ridge basalt (MORB) [2]. This observation suggests that LLSVPs may have originated from an early basal magma ocean (BMO) and could have retained large amounts of water at the present-day core-mantle boundary (CMB, see Supplementary Materials for a brief review). Lu *et al.* recently demonstrated that water can be incorporated into bridgmanite at high temperatures (∼36–71 GPa and ∼3618–4435 K), raising the possibility that lower mantle minerals crystallizing from a magma ocean can be hydrated. During the subsequent solidification of the BMO, the early crystallization of bridgmanite rapidly depletes Si from the silicate melt, producing a ∼1000-km-thick basal layer containing ∼20 wt% ferropericlase (Fp) [4]. Although core-exsolution may mitigate this enrichment [5], seismic tomography still requires 6%–10% Fp at the CMB [6]. Critically, because the elastic properties of ferropericlase significantly reduce seismic velocities, this provides a mechanism for ULVZs [7], while the additional presence of water may further suppress wave speeds. Therefore, quantifying the partitioning of water and iron between ferropericlase and melt during BMO crystallization is essential for understanding both deep-water storage and the origin of these enigmatic low-velocity structures.

However, research into the hydration of ferropericlase is currently limited, focusing primarily on its water solubility, with available data being both scarce and contradictory. Previous experiments at 25.5 GPa suggested that water content could reach up to 2000 ppm based on Secondary Ion Mass Spectrometry (SIMS) measurements [8]. Subsequent studies using Fourier-transform infrared spectroscopy (FTIR) revealed water solubility in ferropericlase two orders of magnitude lower [9]. This discrepancy arises because SIMS is prone to overestimation due to its inability to effectively exclude microscopic hydrous inclusions, whereas FTIR provides a more accurate measurement of structurally bound water. Consequently, FTIR has become the preferred standard for such analyses. Nevertheless, because melts or fluids cannot be quenched effectively in these experiments, the partition coefficient $D_{H_2O}^{Fp/Melt}$cannot be directly measured, and values inferred from earlier experiments remain highly questionable. Current FTIR-based results indicate that water solubility in ferropericlase is between 10 and 80 ppm, a range consistent with natural samples but almost two orders of magnitude lower than that of bridgmanite. Furthermore, interstitial melts in ferropericlase are far too small (<200 nm) to allow reliable water measurements, leaving $D_{H_2O}^{Fp/Melt}$still undetermined. Although *ab initio* calculations are able to access the deep mantle pressure–temperature conditions, they are largely restricted to obtaining partition coefficients between pure solid phases in the absence of melt [10].

To address these challenges, we employed a novel approach combining laser-heated Diamond Anvil Cell (DAC) experiments with NanoSIMS analysis, a technique capable of precisely measuring water content in microscopic samples [3,11]. The laser-heated DAC was used to simulate magma ocean conditions and NanoSIMS was employed to quantify the partitioning of water between mineral facies (ferropericlase and bridgmanite) and the coexisting silicate melt. Conventional techniques typically use a focused ion beam to cut a vertical cross-section of the DAC sample within its gasket. However, this often yields to an analytical area too small for reliable NanoSIMS measurements. To overcome this limitation, we implemented a manual polishing technique in which the sample is polished parallel to the diamond culet surface. This exposes a significantly larger surface area, thereby enhancing measurement accuracy and reliability [11]. (see Supplementary Materials for Supplementary Methods and Fig. S1).

Our experiments were carried out at pressures ranging from 35 to 40 GPa, and temperatures between 3000 and 4000 K. The results reveal that water partitions strongly into the silicate melt, which contains ∼18 000 ppm H_2_O. Bridgmanite incorporated about 600–1000 ppm of water, yielding a partition coefficient $D_{{H}_2O}^{Brg/Melt}\ \approx \ 0.055{-}0.065$ (see Supplementary Materials for Table S1). This finding is in great agreement with the recent results of Lu *et al*. Most strikingly, ferropericlase was found to contain ∼3500 ppm H_2_O, yielding a partition coefficient $D_{{H}_2O}^{Fp/Melt}\ \approx \ 0.205$. This water concentration far exceeds the previously established solubility limit of around 80 ppm. These results challenge the long-held notion that water is highly incompatible in ferropericlase, suggesting instead that under magma ocean conditions, ferropericlase is a significantly more important water host than bridgmanite.

Bridgmanite and ferropericlase are the primary crystallization products of a primordial magma ocean (MO). As the MO solidifies, water preferentially partitions into the residual melt. *Ab initio* calculations indicate that this hydrous residual melt becomes denser than the coexisting crystalline phases, ensuring its gravitational stability at the base of the mantle [12]. This process would logically culminate in the formation of a highly hydrous BMO during the final stages of global crystallization. According to our newly determined partition coefficient ($D_{{H}_2O}^{Fp/Melt}$), ferropericlase crystallizing from this water-rich BMO would be significantly enriched in water. The high density of this hydrous ferropericlase would allow it to remain stable at the base of the lower mantle over geological timescales [13], potentially forming a distinct, water-rich layer at the CMB. We propose that this deep, hydrated material could be entrained into mantle plumes and transported to the surface, manifesting as OIBs. This mechanism provides a compelling explanation for the persistent geochemical observation that OIB source regions consistently contain more water than MORB sources (Fig. 1).

**Figure 1.**
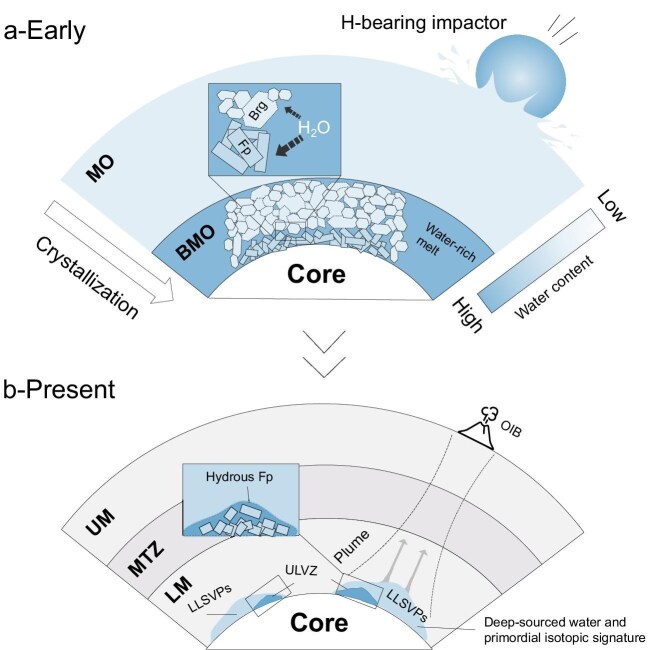
Schematic evolution of deep mantle low-velocity anomalies and primordial water reservoirs. (a) During the crystallization of the early-Earth magma ocean, a water-rich basal magma ocean (BMO) formed. As the BMO continued to crystallize, water was extensively incorporated into ferropericlase and bridgmanite, preferentially partitioning into ferropericlase, thereby producing hydrous ferropericlase. The ferropericlase distributed at the core-mantle boundary (CMB) [5,6] constitutes a primordial water reservoir. (b) Over geological time, this layer evolves into the ultra-low velocity zones (ULVZs) observed today and potentially contributes to the formation of large low-shear-velocity provinces (LLSVPs). This deep, ancient reservoir is hypothesized to be the source of water-rich ocean-island basalts (OIBs), which are sampled and transported upward by mantle plumes.

Previous studies demonstrate that if ULVZs are enriched in ferropericlase, their geophysical characteristics can be explained self-consistently [14]. In this scenario, ULVZs represent not only relics of planetary differentiation but also significant reservoirs of primordial water. The incorporation of substantial water into the ferropericlase lattice is expected to significantly reduce its shear modulus, potentially providing a direct physical mechanism for the extreme seismic velocity reductions observed in ULVZs. Building on this, we propose that the seismic velocity variations among different LLSVPs and ULVZs may reflect regional differences in the proportion of ferropericlase formed during the solidification of the BMO. This offers a complementary mechanism for generating large-scale chemical and hydrological heterogeneity in the deep mantle. Nevertheless, quantifying the total mass of water-bearing ferropericlase crystallized and preserved during the BMO stage remains a critical challenge. Further dedicated experimental and theoretical studies are required to fully constrain its role in Earth’s long-term evolution.

As we continue to push the boundaries of experimental capabilities, the development of innovative sample preparation techniques becomes increasingly crucial. Manual polishing of high-pressure DAC ferropericlase samples, especially those above 40 GPa, is currently a major bottleneck in obtaining testable samples. Consequently, the experimental data presented here should be regarded as preliminary. While our experiments were conducted under water-saturated conditions, thermodynamic constraints suggest that common discrete hydrous phases are typically unstable under our peak P-T conditions (∼3000–4000 K, ∼35–40 GPa; see Supplementary Materials for Influence of hydrous phase inclusions). However, we explicitly acknowledge that our current NanoSIMS measurements cannot unambiguously differentiate structurally incorporated water from potential contributions arising from nanoscale inclusions, grain boundaries, or secondary phases. Because the ferropericlase grains crystallized at 40 GPa are extremely minute—significantly smaller than those of coexisting bridgmanite—verifying the absence of such nanoscale features using independent spectroscopic constraints (e.g. FTIR or Raman) remains technically unfeasible at present. Therefore, while these data represent the highest-pressure constraints currently achievable, resolving the precise 3D distribution of hydrogen and fully reconciling SIMS measurements with spectroscopic estimates will require the development of more advanced, higher-resolution instrumentation. Furthermore, a critical unresolved question concerns the density contrast between hydrous ferropericlase, and the coexisting silicate melt under BMO conditions. Determining this relationship is essential, as it dictates whether early-crystallizing ferropericlase is dense enough to accumulate and persist at the base of the mantle. Addressing this gravitational stability criteria represents a priority for future experimental investigation.

## Supplementary Material

nwag258_Supplemental_File
