# Supplementary material for: From magma ocean to core–mantle boundary: the key role of hydrous ferropericlase in shaping deep Earth’s low-velocity anomalies
Source: Natl Sci Rev. 2026 May 1;13(9):nwag258. doi: 10.1093/nsr/nwag258 (PMC13188978; doi:10.1093/nsr/nwag258)
Supplement: nwag258_Supplemental_File [file nwag258_supplemental_file.docx]

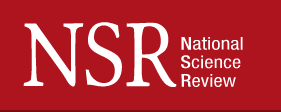


**Supplementary Information for**

**From Magma Ocean to Core–Mantle Boundary: The Key Role of Hydrous Ferropericlase in Shaping Deep Earth’s Low-Velocity Anomalies**

Shi-Dong Guan^1,2^, Yu Wang^1*^, Zhi-Xue Du^1^, Ya-Nan Yang^1, 3^, Yi-Gang Xu^1^

^1^State Key Laboratory of Deep Earth Processes and Resources, Guangzhou Institute of Geochemistry, Chinese Academy of Sciences, Guangzhou 510640, China

^2^College of Earth and Planetary Sciences, University of Chinese Academy of Sciences, Beijing 100049, China

^3^Department of Chemistry and Molecular Biology, University of Gothenburg, Gothenburg 41296, Sweden

^*^Correspondences. Email: wangyu@gig.ac.cn

# This PDF file includes:

Supplementary Texts

Supplementary Methods

Supplementary Figures

Supplementary Table

Supplementary References

# Supplementary Texts

**Brief Review**

At Earth’s core–mantle boundary lies two massive seismic (low-velocity) anomalies: the Large Low Shear Velocity Provinces (LLSVPs) beneath Africa and the Pacific, and the smaller Ultra-Low Velocity Zones (ULVZs) scattered within or along their margins [1-2]. These anomalies exhibit moderate reductions in compressional (V_P_) and shear (V_S_) wave speeds, virtually unchanged bulk-sound velocity (V_Φ_), sharp boundaries and other distinct seismic signatures [3]. Geochemically, Ocean Island Basalts (OIBs) differ significantly from the Mid-Ocean Ridge Basalts (MORBs) that characterize the upper mantle [4]. OIBs carry distinct isotopic fingerprints and trace-element anomalies indicative of ancient source regions, suggesting a genetic link to the deep-seated LLSVPs [5-10]. Recent studies propose that LLSVPs and ULVZs originated from differentiation of a basal magma ocean (BMO) [11-13]. Following the high-energy Moon-forming impact, a global magma ocean would have crystallized such that iron preferentially partitioned into the melt, producing a dense, negatively buoyant silicate melt layer at the mantle base [14-18]. Consequently, the anomalous geochemical signals preserved in OIBs may hold the key to unravelling the enigmatic nature of LLSVPs and ULVZs at the core–mantle boundary.

**Phase identification for NanoSIMS analyses**

We first determined the approximate compositions of the two phases using a BSE image (Fig. S1b) and EDS (Table. S1). They were further distinguished in NanoSIMS maps based on their distinct elemental signatures (Fig. S1c): ferropericlase [(Mg,Fe)O] is characterized by low Si and high Mg contents, whereas bridgmanite [(Mg,Fe)SiO_3_] shows high Si and moderate Mg concentrations.

**Influence of hydrous phase inclusions**

While our experiments were conducted under water-saturated conditions, thermodynamic constraints suggest that common discrete hydrous phases are typically unstable under our peak P-T conditions (~3000-4000 K, ~35-40 GPa). For instance, brucite is generally stable only below 35 GPa [19], and phase D and the δ-H phase are restricted to temperatures below ~2500 K [20] and ~2190 K [21], respectively. We acknowledge that large temperature gradients are ubiquitous in laser-heated LHDAC experiments. Without further verification via high-precision analytical methods, the potential influence of secondary hydrous phases on our results cannot be fully ruled out. To address this concern, we performed Raman spectroscopy on ferropericlase domains (Fig. S2) using a WITec alpha 300R Raman microscope equipped with a 532 nm excitation laser and a 100×Zeiss objective (Zeiss EC Epiplan-Neofluar Dic, NA = 0.9), providing a theoretical spatial resolution of ~0.5 μm. A broad Raman band near 3500 cm^-1^ was observed, supporting the presence of structural water within the MgO lattice; however, no discrete hydrous mineral peaks of discrete were detected (Fig. S2). Due to the extremely small sample size (~1 μm) and transparency to the 532 nm laser, clear ferropericlase Raman signals were not obtained, and spectral contributions from surrounding or underlying phases cannot be completely excluded (Fig. S2). Thus, these preliminary Raman results serve as reference data and highlight the need for more refined analytical approaches in future investigations.

# Supplementary Methods

**Laser-heated diamond-anvil cell experiments**

We used 300 um culet diamond anvils. A Re gasket (initial thickness 250 um) was pre-indented to 30－40 um and then laser-drilled to create a 100 um sample chamber. Glass fragments were loaded into the hole and compressed to the target pressure.

Heating was performed with a bilateral, defocused 1064 nm laser under programmable power control. The laser power ramps to its peak within 0.5 s, is held for 0.5–1 s, and is then shut off. Thermal radiation was recorded at 24 frames per second with a four-wavelength (580, 656, 758, 920 nm) two-dimensional temperature-measurement system at the State Key Laboratory of Deep Earth Processes and Resources, Guangzhou Institute of Geochemistry, CAS, similar to Yang et al.,(2023). The last thermal-radiation frame before quench was used to determine the experimental temperature.

Pressure was measured by diamond Raman spectroscopy [22] before and after heating and corrected for thermal pressure using ΔP = 2.7 MPa/K [23]. After decompression, samples were mounted in epoxy and hand-polished parallel to the surface with Al_2_O_3_ sandpaper to obtain a flat section for subsequent chemical analysis. (Fig. S1)

**NanoSIMS analysis**

Polished specimens were removed from the epoxy mount and re-attached to a steel holder. To minimize atmospheric water contamination and reduce instrumental background, both samples and standards were dried in a vacuum oven at 323 K for two weeks, then sputter-coated with gold before loading into the NanoSIMS. Prolonged heating at 323 K should be avoided, however, to prevent diffusive hydrogen loss before or after analysis, especially when water is present as molecular H_2_ in glass or minerals. Water contents of bridgmanite, ferropericlase and silicate glass were determined with a CAMECA NanoSIMS 50L at the State Key Laboratory of Deep Earth Processes and Resources, Guangzhou Institute of Geochemistry, Chinese Academy of Sciences, following the analytical protocol of Yang et al. (2023) and Lu et al. (2025).

**Supplementary Figure**


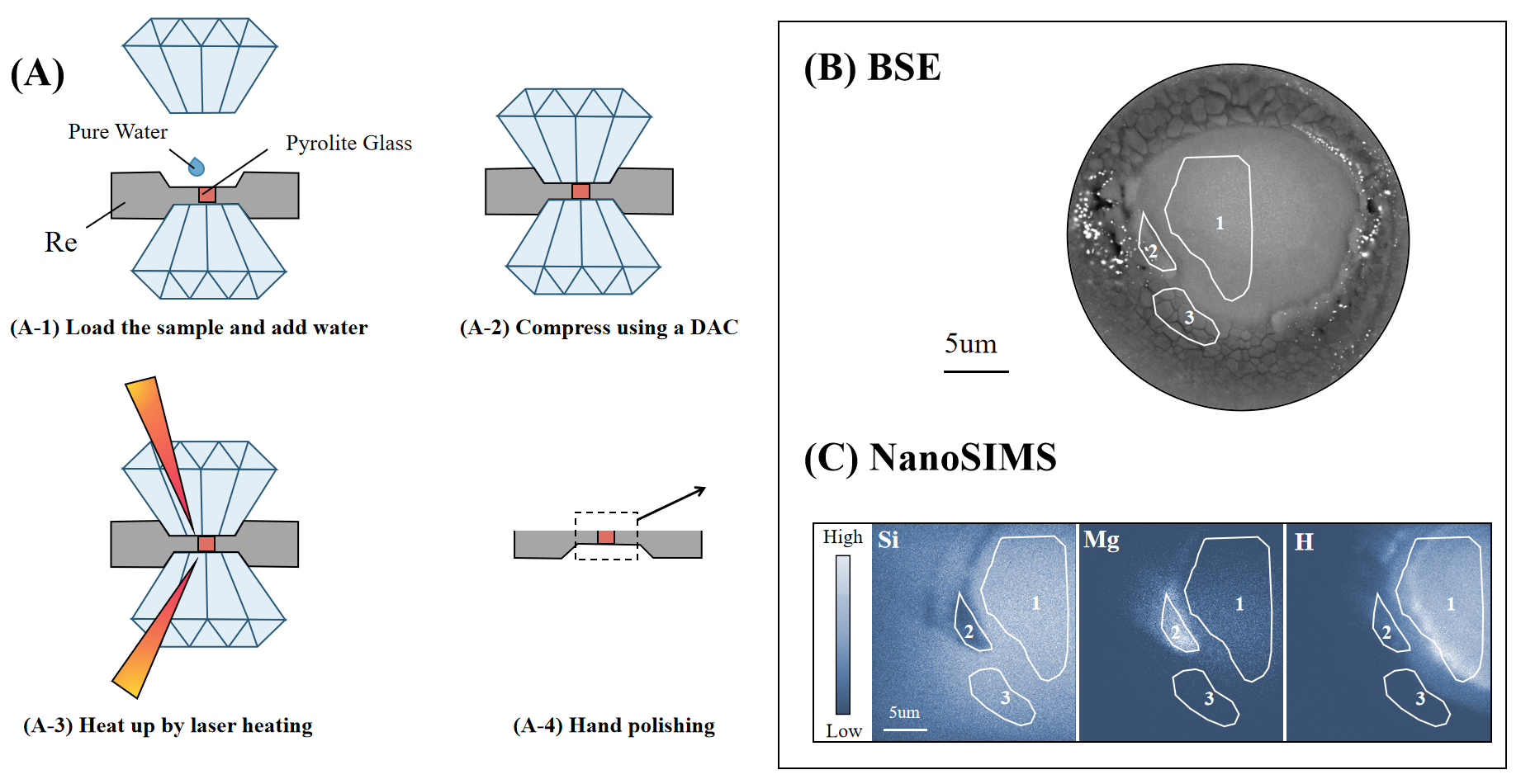


**Fig. S1 Experimental evidence for hydrous ferropericlase.** (A) Sample preparation procedure Backscattered. (B) Backscattered Electron (BSE) image of the experimental charge recovered from the DAC after laser heating. This image reveals the texture of the coexisting phases. (C) Corresponding NanoSIMS mapping of the DAC heating area, consisting of three images showing the distribution of Si (identifying bridgmanite), Mg (identifying ferropericlase), and H (determining the water content in various mineral phases and melts). 1-Melt, 2-Ferropericlase and 3- Bridgmanite.


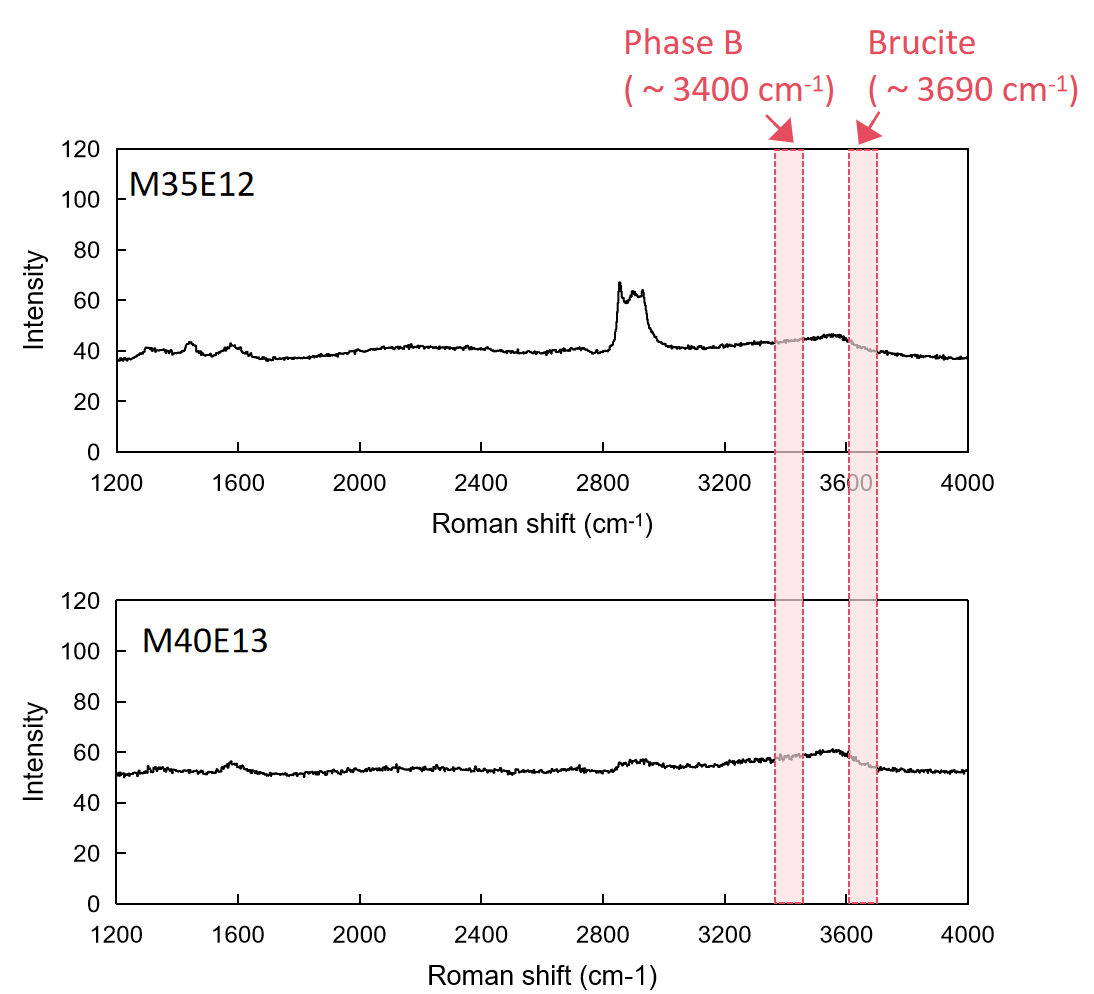


**Fig. S2 Raman spectra of the recovered ferropericlase phases region.** Peaks at ~3,400 or 3,690 cm-1, typically associated with hydrous phases inclusions. Note: the Raman results obtained in this study are for reference only, with detailed discussion provided in the above section Influence of hydrous phase inclusions.

**Supplementary Table**

| **Table S1. Chemical compositions of the recovered samples.** | | | | | | |
| --- | --- | --- | --- | --- | --- | --- |
| **Run No.** | **M35E12** | | | **M40E13** | | |
| P (GPa) | 35 (2) | | | 40 (3) | | |
| T (K) | 3007 (127) | | | 3300 (213) | | |
| Phase | Fp | Brg | Melt | Fp | Brg | Melt |
| MgO | 62.12 | 37.05 | 29.37 | 73.59 | 36.63 | 36.67 |
| Al_2_O_3_ | 3.25 | 5.14 | 5.76 | 0 | 6.86 | 6.06 |
| SiO_2_ | 14.88 | 52.91 | 42.04 | 10.38 | 51.07 | 41.51 |
| CaO | 1.43 | 1.85 | 6.33 | 1.43 | 2.45 | 4.03 |
| FeO_T_ | 17.23 | 3.34 | 15.17 | 15.41 | 4.23 | 10.18 |
| H_2_O (ppm) | 3391 (175) | 1077 (89) | 16447 (1541) | 3982 (208) | 660 (63) | 16132 (2132) |
| Total | 99.25 | 100.40 | 100.31 | 101.21 | 101.31 | 100.06 |

**Note:** the major-element data are EDS results, intended only for phase identification. The water content data are the results of NanoSIMS. FeO_T_ means total iron. Parentheses denote uncertainties.

# Supplementary References

1. Garnero, E. J., McNamara, A. K. & Shim, S.-H. (2016). Continent-sized anomalous zones with low seismic velocity at the base of Earth’s mantle. Nat. Geosci. ***9***, 481–489 .
2. Hedlin, M. A. H., Shearer, P. M. & Earle, P. S. (1997). Seismic evidence for small-scale heterogeneity throughout the Earth’s mantle. Nature ***387***, 145–150 .
3. Trampert, J., Deschamps, F., Resovsky, J. & Yuen, D. (2004). Probabilistic tomography maps chemical heterogeneities throughout the lower mantle. Science ***306***, 853–856 .
4. Hofmann, A. W. & Hart, S. R. (1978). An assessment of local and regional isotopic equilibrium in the mantle. Earth Planet. Sci. Lett. ***38***, 44–62 .
5. Pringle, E. A. et al. (2016). Silicon isotopes reveal recycled altered oceanic crust in the mantle sources of ocean island basalts. Geochim. Cosmochim. Acta ***189***, 282–295 .
6. Deng, J. & Stixrude, L. (2021). Deep fractionation of Hf in a solidifying magma ocean and its implications for tungsten isotopic heterogeneities in the mantle. Earth Planet. Sci. Lett. ***562***, 116873 .
7. Mukhopadhyay, S. & Parai, R. (2019). Noble gases: a record of Earth’s evolution and mantle dynamics. Annu. Rev. Earth Planet. Sci. ***47***, 389–419.
8. Mundl-Petermeier, A. et al. (2020). Anomalous 182W in high 3He/4He ocean island basalts: fingerprints of Earth’s core? Geochim. Cosmochim. Acta <https://doi.org/10.1016/j.gca.2019.12.020> .
9. Niu, Y. (2018). Origin of the LLSVPs at the base of the mantle is a consequence of plate tectonics—a petrological and geochemical perspective. Geosci. Front.***9***, 1265–1278 .
10. Rizo, H. et al. (2019). 182W evidence for core–mantle interaction in the source of mantle plumes. Geochem. Perspect. Lett. ***11***, 6–11.
11. Deschamps, F., Kaminski, E. & Tackley, P. J. (2011). A deep mantle origin for the primitive signature of ocean island basalt. Nat. Geosci. ***4***, 879–882 .
12. Labrosse, S., Hernlund, J. W. & Coltice, N. (2007). A crystallizing dense magma ocean at the base of the Earth’s mantle. Nature ***450***, 866–869 .
13. Pachhai, S., Li, M., Thorne, M. S., Dettmer, J. & Tkalčić, H. (2022). Internal structure of ultralow-velocity zones consistent with origin from a basal magma ocean. Nat. Geosci. ***15***, 79–84.
14. Nomura, R. et al. (2011). Spin crossover and iron-rich silicate melt in the Earth’s deep mantle. Nature ***473***, 199–202 .
15. Caracas, R., Hirose, K., Nomura, R. & Ballmer, M. D. (2019). Melt–crystal density crossover in a deep magma ocean. Earth Planet. Sci. Lett. ***516***, 202–211.
16. Miyazaki, Y. & Korenaga, J. (2019). On the timescale of magma ocean solidification and its chemical consequences: 2. Compositional differentiation under crystal accumulation and matrix compaction. J. Geophys. Res. ***124***, 3399–3419.
17. Boukaré, C.-É., Badro, J. & Samuel, H. (2025). Solidification of Earth’s mantle led inevitably to a basal magma ocean. Nature ***640***, 114–119 .
18. Stixrude, L., Scipioni, R. & Desjarlais, M. P. (2020). A silicate dynamo in the early Earth. Nat. Commun. ***11***, 935 .
19. A. Hermann, M. Mookherjee. (2016). High-pressure phase of brucite stable at Earth’s mantle transition zone and lower mantle conditions. Proceedings of the National Academy of Sciences ***113***, 13971–13976 .
20. Y. Duan, N. Sun, S. Wang, X. Li, X. Guo, H. Ni, V. B. Prakapenka, Z. Mao. (2018) Phase stability and thermal equation of state of δ-AlOOH: Implication for water transportation to the Deep Lower Mantle. Earth and Planetary Science Letters ***494***, 92–98 .
21. I. Ohira, E. Ohtani, T. Sakai, M. Miyahara, N. Hirao, Y. Ohishi, M. Nishijima. (2014). Stability of a hydrous δ-phase, AlOOH–MgSiO_2_(OH)_2_, and a mechanism for water transport into the base of lower mantle. Earth and Planetary Science Letters ***401***, 12–17.
22. Y. Akahama, H. Kawamura. (2006). Pressure calibration of diamond anvil Raman gauge to 310GPa. Journal of Applied Physics ***100***, 043516.
23. G. Fiquet, A. L. Auzende, J et al. (2010). Melting of Peridotite to 140 Gigapascals. Science ***329***, 1516–1518
